# Identification and Characterization of a Small Molecule Bcl-2 Functional Converter

**DOI:** 10.1158/2767-9764.CRC-22-0526

**Published:** 2024-03-04

**Authors:** Prasad R. Kopparapu, Martin C. Pearce, Christiane V. Löhr, Cathy Duong, Hyo Sang Jang, Shanthakumar Tyavanagimatt, Edmond F. O'Donnell, Harikrishna Nakshatri, Siva K. Kolluri

**Affiliations:** 1 Cancer Research Laboratory, Department of Environmental and Molecular Toxicology, Oregon State University, Corvallis, Oregon.; 2 Department of Biomedical Sciences, Carlson College of Veterinary Medicine, Oregon State University, Corvallis, Oregon.; 3 Department of Surgery, Indiana University School of Medicine, Indianapolis, Indiana.; 4 Linus Pauling Institute, Oregon State University, Corvallis, Oregon.

## Abstract

Cancer cells exploit the expression of anti-apoptotic protein Bcl-2 to evade apoptosis and develop resistance to therapeutics. High levels of Bcl-2 leads to sequestration of pro-apoptotic proteins causing the apoptotic machinery to halt. In this study, we report discovery of a small molecule, BFC1108 (5-chloro-N-(2-ethoxyphenyl)-2-[(4-methoxybenzyol)amino]benzamide), which targets Bcl-2 and converts it into a pro-apoptotic protein. The apoptotic effect of BFC1108 is not inhibited, but rather potentiated, by Bcl-2 overexpression. BFC1108 induces a conformational change in Bcl-2, resulting in the exposure of its BH3 domain both *in vitro* and *in vivo*. BFC1108 suppresses the growth of triple-negative breast cancer xenografts with high Bcl-2 expression and inhibits breast cancer lung metastasis. This study demonstrates a novel approach to targeting Bcl-2 using BFC1108, a small molecule Bcl-2 functional converter that effectively induces apoptosis in Bcl-2–expressing cancers.

**Significance:**

We report the identification of a small molecule that exposes the Bcl-2 killer conformation and induces death in Bcl-2–expressing cancer cells. Selective targeting of Bcl-2 and elimination of cancer cells expressing Bcl-2 opens up new therapeutic avenues.

## Introduction

Bcl-2-family proteins are evolutionarily conserved regulators of apoptosis. All members possess at least one of the four conserved motifs called Bcl-2 homology (BH) domains. The anti-apoptotic Bcl-2 family members consist of Bcl-2, Bcl-xL, Mcl-1, AI-1, Bcl-B and contain BH domains 1–4. There are two groups of pro-apoptotic Bcl-2 members; the BH1–3 containing group such as Bax and Bak, or the BH3 only group such as Bid, Bim, and Puma (1–6). The BH3 only pro-apoptotic members function by activating multi-domain pro-apoptotic proteins like Bax and Bak (7–9). The anti-apoptotic Bcl-2 members use the hydrophobic surface binding pocket formed by the BH 1–3 domains to neutralize these pro-apoptotic proteins. It is the ratio of the levels of these anti-apoptotic and pro-apoptotic proteins that cancer cells exploit for their survival advantage (6). Specifically, Bcl-2 overexpression leads to cancer progression and resistance to cancer therapies (10–13).

The Bcl-2 family of proteins is being pursued with multiple approaches to achieve therapeutic outcomes (14–19). Small molecules like ABT-737 or ABT-199 are being developed to either inhibit multiple anti-apoptotic members or a single anti-apoptotic member (20–22). These inhibitors are designed to bind to the hydrophobic groove formed by the BH1–3 domains of anti-apoptotic members (23–25). NuBCP-9, a 9 amino acid peptide derived from nuclear receptor Nur77 binds the loop domain in Bcl-2, resulting in exposure of its BH3 domain and conversion of Bcl-2 into a pro-death protein (26–41). We hypothesized that small molecules targeting the Bcl-2 that switch its function to a pro-death protein can be developed to effectively treat cancers that express Bcl-2.

To test this hypothesis, we screened the Chembridge DIVERset® library using MDA-MB-231 cells stably expressing Bcl-2 along with their empty vector controls that have very low expression of Bcl-2 in a viability assay. Compounds were shortlisted on the basis of their ability to inhibit viability of Bcl-2–expressing cancer cells, compared with their control cells. These shortlisted compounds were further probed for their ability to induce a conformational change in Bcl-2 and promote Bcl-2–dependent apoptosis. One such compound, termed here as Bcl-2 functional converter (BFC)1108 (5-chloro-N-(2-ethoxyphenyl)-2-[(4-methoxybenzyol)amino]benzamide), induced a conformational change in Bcl-2, resulting in Bcl-2–dependent cell death. The anticancer effects of BFC1108 were further tested in a breast cancer xenograft mouse model using MDA-MB-231 cells expressing Bcl-2 and in a breast cancer lung metastatic model.

## Materials and Methods

### Cell Culture

MDA-MB-231 (ATCC, catalog no. CRM-HTB-26, RRID:CVCL_0062), MCF-7 (ATCC, catalog no. HTB-22, RRID:CVCL_0031), HepG2 (ATCC, catalog no. HB-8065, RRID:CVCL_0027), ZR-75-1 (ATCC, catalog no. CRL-1500, RRID:CVCL_0588), A375 (ATCC, catalog no. CRL-1619, RRID:CVCL_0132), mouse embryonic fibroblasts (MEF; ATCC, catalog no. CRL-2991, RRID:CVCL_U630), and H460 (ATCC, catalog no. HTB-177, RRID:CVCL_0459) were cultured in DMEM with l-glutamine (Corning, catalog no. 10-013-CV) supplemented with 10% FBS (tissue culture biologicals, catalog no. 101 HI) with 10,000 U/mL penicillin streptomycin (Corning, catalog no. 30-002-Cl) in a humidified chamber with 5% CO_2_. LNCaP cells (ATCC, catalog no. CRL-1740, RRID:CVCL_0395) were cultured in RPMI1640 (Corning, catalog no. 10-040-CV). MCF-10A cells (ATCC, catalog no. CRL-10317, RRID:CVCL_0598) were cultured in DMEM/F-12 (Gibco, catalog no. 11-320-033) with appropriate supplements. All cells were passaged once every 3 days.

### Generation of Stable Cell Lines

MDA-MB-231 and MCF-7 breast cancer cell lines stably overexpressing Bcl-2 were generated via electroporation and subsequent clonal expansion in G418-containing media. MDA-MB-231 cell clones with sufficiently high expression of Bcl-2 (MDA-MB-231/Bcl-2) were determined by Western blotting. These were utilized for chemical library screening along with their vector controls (MDA-MB-231/Vector). Similarly, MCF-7 cell clones were derived and were utilized for characterization of lead molecules.

### Chemicals

The DIVERset® chemical library was purchased from ChemBridge Corporation. All compound dilutions were made in DMSO. BFC1108 (5-chloro-N-(2-ethoxyphenyl)-2-[(4-methoxybenzyol)amino]benzamide) was custom synthesized by ChemBridge Corporation.

### Viability Assays

CellTiter-Glo assay (Promega, catalog no. G7573) was used for assessing viability (42, 43). Annexin V (eBioscience, catalog no. 88-8008) was used to assess apoptosis (44, 45).

### Antibodies

Bcl-2 BH3 antibody (catalog no. AP1303a) was obtained from Abgent. Bcl-2 antibody (catalog no. 13-8800) was obtained from InvitrogenCA. Ki-67 and hematoxylin and eosin (H&E) was obtained from Cell Signaling Technology. JC-1 dye (catalog no. T3168) was obtained from Roche. Western Blotting, immunofluorescence, and IHC experiments were done as described in (refs. 26–28, 35).

### Bcl-2 Knockdown

For Bcl-2 knockdown experiments, MDA-MB-468 cells were seeded at 4,000 cells per well in a 96-well black plate. The day after seeding, cells were transfected using Dharmafect reagent (catalog no. T-2001-03) with Bcl-2 siRNA (25 ng; Dharmacon Research) or 25 ng control luciferase siRNA (catalog no. D-002050-01, Dharmacon Research). After 72 hours of transfection, cells were treated with indicated compounds for 48 hours and viability was assayed with CellTiter-Glo Luminescent Cell Viability Assay (Promega, catalog no. G7573).

### Limited Proteolysis Assay

GST-Bcl-2 and GST only fragments (60 µg) were incubated with 0.1% DMSO or BFC1108 (50 µmol/L) for 1 hour and proteolyzed by 3 µg/mL of trypsin for 1, 2, 4, and 8 minutes. Reactions were quenched by the addition of Laemmli buffer. The resulting protease cleavage fragments were resolved on a 12% SDS-PAGE gel and visualized by Coomassie blue staining.

### Protein Thermal Shift Assay

Bcl-2 protein was mixed with elution buffer and SYPRO Orange dye and then mixed with BFC1108 at varying concentrations. Samples were loaded into PCR plate and covered. A 7500 Fast PCR system (Applied Biosystems) was used with detection set to ROX and initial 2-minute incubation at 25°C followed by step and hold 0.5°C for 30 seconds up until 95°C. Melting temperature was calculated using Applied Biosystems software (46).

### Animal Experiments

All animal experiments were performed as per approved protocol no. 4452 by the Institutional Animal Care and Use Committee (IACUC) at Oregon State University (Corvallis, OR). For the mammary fat pad xenograft study, 5-week-old NOD.SCID (RRID:IMSR_ JAX:001303) mice were procured from Jackson labs. About 10^6^ cells were injected into both the lower flanks of mammary fat pad in PBS. Palpable tumors were detected in 4 weeks after which mice were treated with BFC1108 (100 mg/kg) twice a week till end of the study (*N* = 8 for vehicle and BFC1108 treatments). Tumor mass was measured using digital calipers twice a week along with body weight. At the end of the study, mice were euthanized and tumors were collected for IHC analysis.

For the lung metastatic study, 5-week-old Nude mice (RRID:IMSR_JAX:002019) were procured from Jackson Labs. About 200,000 LMD231-Luc2 cells in PBS were injected into the tail vein. Lung metastasis was detected by 2 weeks. Treatments with BFC1108 at 100 mg/kg, four times a week, were started a day after the cells were injected (*N* = 8 for vehicle and *N* = 9 for BFC1108 treatment). Body weight was measured twice a week. Bioluminescence was measured once a week while the mice are under anesthesia. At the end of the study, mice were euthanized and lung tissues were dissected and fixed for IHC analysis.

### Statistical Analysis

The statistical significance of difference between groups was analyzed by two-sided unpaired nonparametric Student *t* test with Mann–Whitney test using PRISM software version 9. Results were considered significant at *P* < 0.05.

### Data Availability Statement

The data generated in this study are available within the article and its Supplementary Data.

## Results

### Screening for Small Molecule Bcl-2 Functional Converters

**FIGURE 1:**
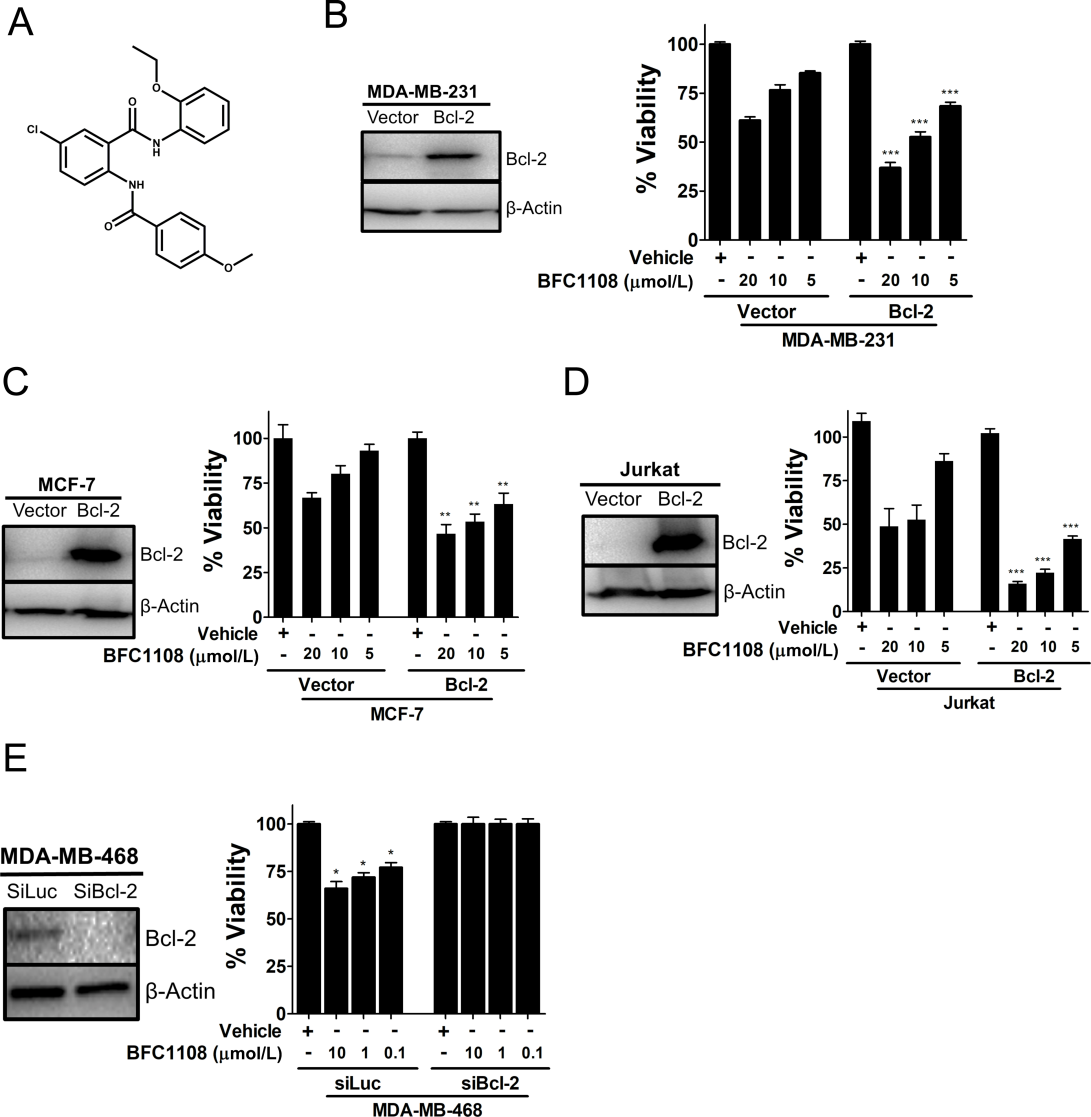
Bcl-2–dependent effects of BFC1108. **A,** Structure of BFC1108 (5-chloro-N-(2-ethoxyphenyl)-2-[(4-methoxybenzyol)amino]benzamide). **B,** Left: Bcl-2 expression in MDA-MB-231 cells transfected with pcDNA control vector (MDA-MB-231/Vector) or Bcl-2 expression vector (MDA-MB-231/Bcl-2) was determined by immunoblotting. Right: Cells were exposed to BFC1108 in a medium containing 10% FBS for 48 hours and viability was determined. ***, *P* < 0.001. **C,** Left: Bcl-2 expression in MCF-7 cells transfected with pcDNA control vector (MCF-7/Vector) or Bcl-2 expression vector (MCF-7/Bcl-2) was determined by immunoblotting. Right: Cells were exposed to BFC1108 in a medium containing 10% FBS for 48 hours and viability was determined. **, *P* < 0.01. **D,** Left: Bcl-2 expression in Jurkat cells transfected with control vector (Jurkat/Vector) or Bcl-2 expression vector (Jurkat/Bcl-2) was determined by immunoblotting. Right: Cells were exposed to BFC1108 in a medium containing 10% FBS for 48 hours and viability was determined. ***, *P* < 0.001. **E,** Left: Knockdown of Bcl-2 in MDA-MB-468 cells was determined by immunoblotting. Right: Bcl-2 siRNA and control siLuc siRNA transfected MDA-MB-468 cells were treated with BFC1108 in a medium containing 10% FBS for 48 hours. *, *P* < 0.05.

To identify small molecules that induce apoptosis in Bcl-2–expressing cancer cells, MDA-MB-231 triple-negative breast cancer (TNBC) cells stably expressing Bcl-2 (MDA-MB-231/Bcl-2) along with its vector controls (MDA-MB-231/Vector) were generated. These cells were used in a viability assay to screen ChemBridge DIVERset® library of compounds. Promising hits were shortlisted and retested for Bcl-2–dependent growth inhibitory effects. A lead compound, termed here as BFC1108 (Fig. 1A) was identified, which dose dependently reduced the viability of Bcl-2–expressing cells (MDA-MB-231/Bcl-2) compared with their vector controls (Fig. 1B). We tested to confirm whether this phenomenon is also observed in other cancer cell types. MCF-7 breast cancer cells with high Bcl-2 expression were more sensitive to BFC1108 compared with their vector controls (Fig. 1C). Similarly, Jurkat T-cell lymphocytes with high Bcl-2 expression were more responsive to BFC1108 with significant reduction in viability compared with their vector controls (Fig. 1D). We next tested the Bcl-2–dependent effect of BFC1108 in a second TNBC MDA-MB-468 cell line. Bcl-2 expression was suppressed in MDA-MB-468 cells by siRNA. BFC1108 reduced the viability of control siRNA-transfected cells in a dose-dependent manner. In contrast, Bcl-2 knocked down MDA-MB-468 cells were resistant to BFC1108 (Fig. 1E). Thus, the effects of BFC1108 are dependent on Bcl-2 expression in distinct cancer cells.

### Bcl-2–selective Effects of BFC1108 in Multiple Cancer Types

**FIGURE 2:**
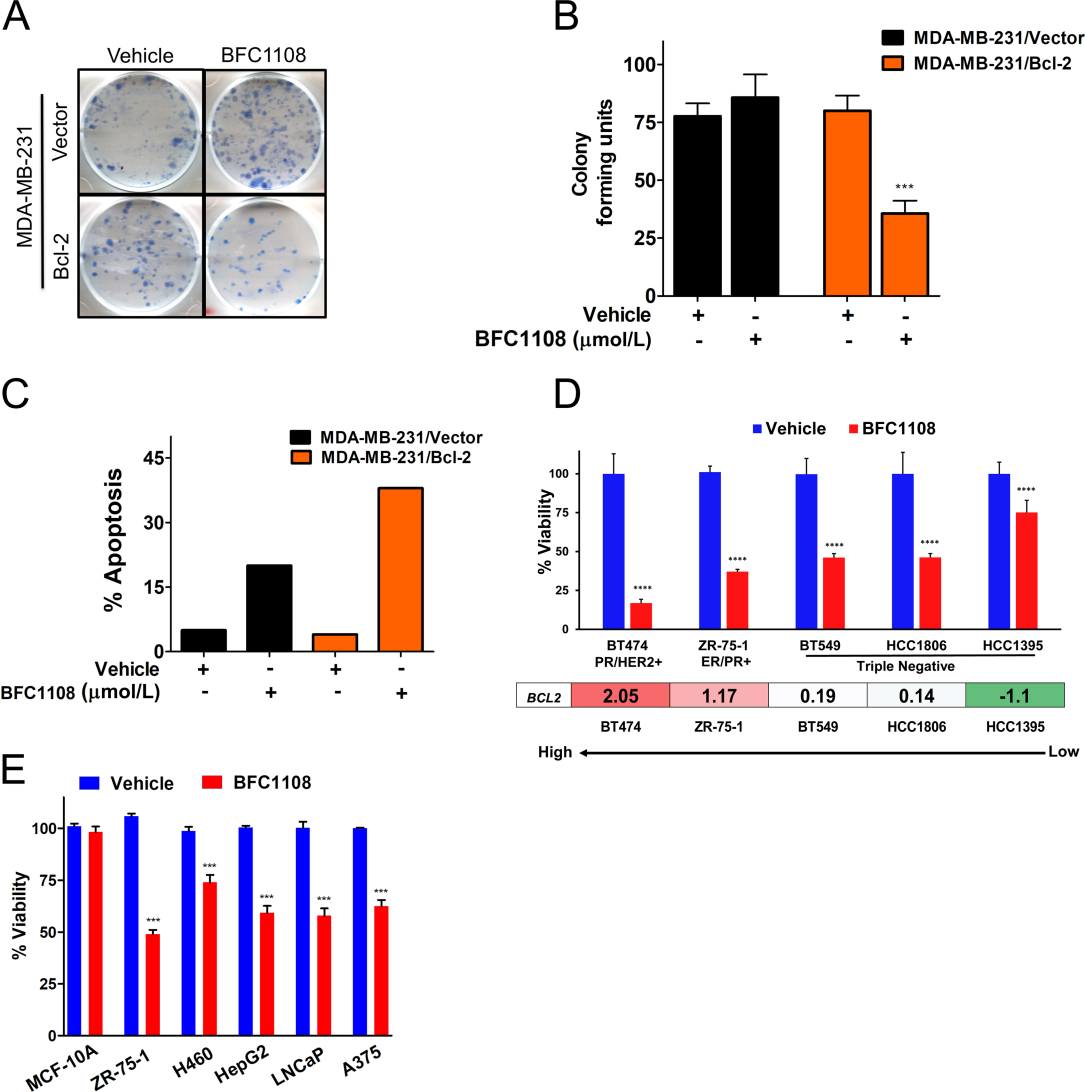
BFC1108 suppresses viability of multiple cancer cell types. **A,** Bcl-2–selective inhibition of clonogenic survival of MDA-MB-231 cells. MDA-MB-231/Vector and MDA-MB-231/Bcl-2 cells were exposed to 10 µmol/L concentration in medium containing 10% FBS for 48 hours and the colony formation was determined after 2 weeks. **B,** Quantification of colony formation data shown in A. ***, *P* < 0.001. **C,** Bcl-2–dependent induction of apoptosis by BFC1108. MDA-MB-231 cells with high or low Bcl-2 expression were treated with 10 µmol/L BFC1108 in a medium containing 10% FBS for 48 hours and apoptosis was determined by annexin V staining. Data from one representative experiment is shown. Refer to Supplementary Fig. S1 for additional independent experiments. **D,** BFC1108 inhibits viability of breast cancer cells with Bcl-2 expression. The indicated breast cancer cells were treated for 48 hours, and viability was measured using CellTiter-Glo assay. Data were analyzed by two-way ANOVA and Dunnett multiple comparison *post hoc* test, **, *P* < 0.001; ****, *P* < 0.00001. Bottom panel indicates Bcl-2 RMA-normalized mRNA expression from the Cancer Cell Line Encyclopedia (50). **E,** BFC1108 suppresses viability of multiple cancer cell types with minimal effects on MCF10A nontransformed breast epithelial cells. Cells were treated for 48 hours in 10% serum containing medium and cell viability was determined. ***, *P* < 0.001.

**FIGURE 3:**
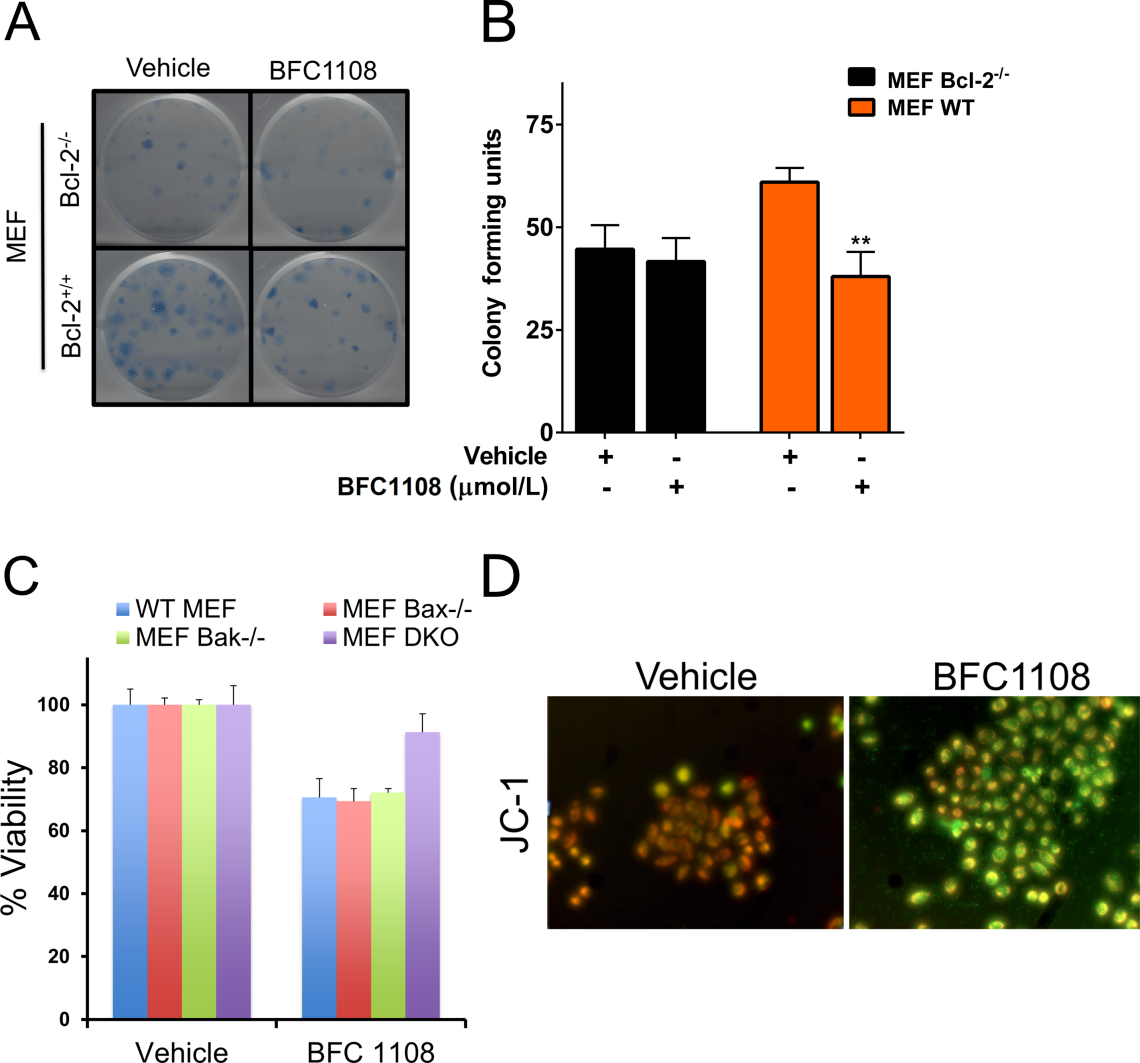
BFC1108 activates intrinsic mitochondrial death pathway. **A,** BFC1108 suppresses the colony-forming ability of MEF cells in a Bcl-2–dependent manner. WT and Bcl-2^−/−^ MEF cells were treated for 48 hours in a medium containing 10% FBS and the colony formation was determined after 2 weeks. **B,** Quantification of colony formation data shown in A. **, *P* < 0.01. **C,** Bax or Bak is required for BFC1108-induced suppression of viability. WT MEF, Bax Knockout (Bax^−/−^ Bak^+/+^), Bak knockout (Bax^+/+^ Bak^−/−^) and double knockout (Bax^−/−^ Bak^−/−^) MEF cells were treated with 1 µmol/L BFC1108 for 24 hours in 10% FBS medium and viability was assessed using CellTiter-Glo assay. **, *P* < 0.01; ***, *P* < 0.001; ns, not significant. **D,** BFC1108 decreases mitochondrial membrane potential of Bcl-2–expressing H460 lung cancer cells. JC-1 dye was used to stain live H460 cells that were treated with 10 µmol/L BFC1108 for 16 hours in 10% FBS containing medium. Images taken with FITC, and rhodamine filters were overlaid. Cells stained orange have intact mitochondrial outer membrane and the ones turning green have compromised outer membrane indicating loss of membrane potential.

We next determined the effect of BFC1108 on clonogenic survival of MDA-MB-231 cells. Upon exposure to BFC1108, MDA-MB-231/Bcl-2 cells formed fewer colonies compared with their vector controls (Fig. 2A and B). Treatment of MDA-MB-231/Bcl-2 cells with 10 µmol/L BFC1108 resulted in a 50% reduction in their colony-forming ability. This effect was not observed in MDA-MB-231/Vector control cells with low Bcl-2 expression. We next tested whether the effect of BFC1108 on cell viability was due to apoptosis. For this, MDA-MB-231/Bcl-2 and their vector control cells were exposed to 10 µmol/L BFC1108 for 48 hours and subsequently stained for annexin V to detect early stages of apoptosis. Similar to the results obtained in viability assays, there was a significant increase in apoptosis of MDA-MB-231/Bcl-2 cells compared with control cells, indicating BFC1108-induced apoptosis depends on Bcl-2 expression (Fig. 2C; Supplementary Fig. S1). The reduction in viability induced by BFC1108 correlated with increased Bcl-2 expression in breast cancer cells (Fig. 2D). Specifically, BT474 cells with higher levels of Bcl-2 expression exhibited a more pronounced response compared with HCC1395 cells, which have lower Bcl-2 expression. Similarly, BT549 and HCC1806, two TNBC cell lines expressing Bcl-2, were responsive to BFC1108 (Fig. 2D). BFC1108 was also effective against a variety of cancer cell types, including human ZR-75-1 hormone receptor–positive breast cancer cells, LNCaP prostate cancer cells, HepG2 hepatocellular carcinoma cells, H460 lung cancer cells, and A375 melanoma cells (Fig. 2E). Notably, BFC1108 exhibited minimal effects on MCF10A nontransformed breast epithelial cells (Fig. 2E). We next tested the effect of BFC1108 on colony-forming ability of transformed MEFs. Wild-type (WT) MEFs formed fewer colonies upon exposure to BFC1108 (10 µmol/L) compared with their vehicle controls (Fig. 3A and B). Bcl-2^−/−^ MEF cells did not respond to BFC1108, indicating a role for Bcl-2 in the reduction of colony-forming units in WT MEFs.

### Requirement of Bax or Bak for BFC1108-induced Effects

To test whether BFC1108 triggers an intrinsic apoptotic pathway, we determined the requirement of Bax and/or Bak in MEFs. BFC1108 reduced the viability of WT (Bax^+/+^ and Bak^+/+^), Bax knockout (Bax^−/−^ Bak^+/+^), and Bak knockout (Bak^−/−^ Bax^+/+^) MEFs. Notably, Bax and Bak double knockout (Bax^−/−^ and Bak^−/−^) MEFs were unaffected by BFC1108 treatment (Fig. 3C). This observation suggests that Bax and/or Bak are essential for the Bcl-2–dependent induction of apoptosis by BFC1108. Furthermore, H460 lung cancer cells which have high levels of endogenous Bcl-2 expression, when treated with BFC1108, exhibited a decrease in mitochondrial membrane potential upon staining with JC-1 dye, indicating the collapse of the mitochondrial outer membrane (Fig. 3D). Thus, the anticancer effects induced by BFC1108 are mediated through an intrinsic mitochondrial cell death pathway.

### Interaction of BFC1108 with Bcl-2

**FIGURE 4:**
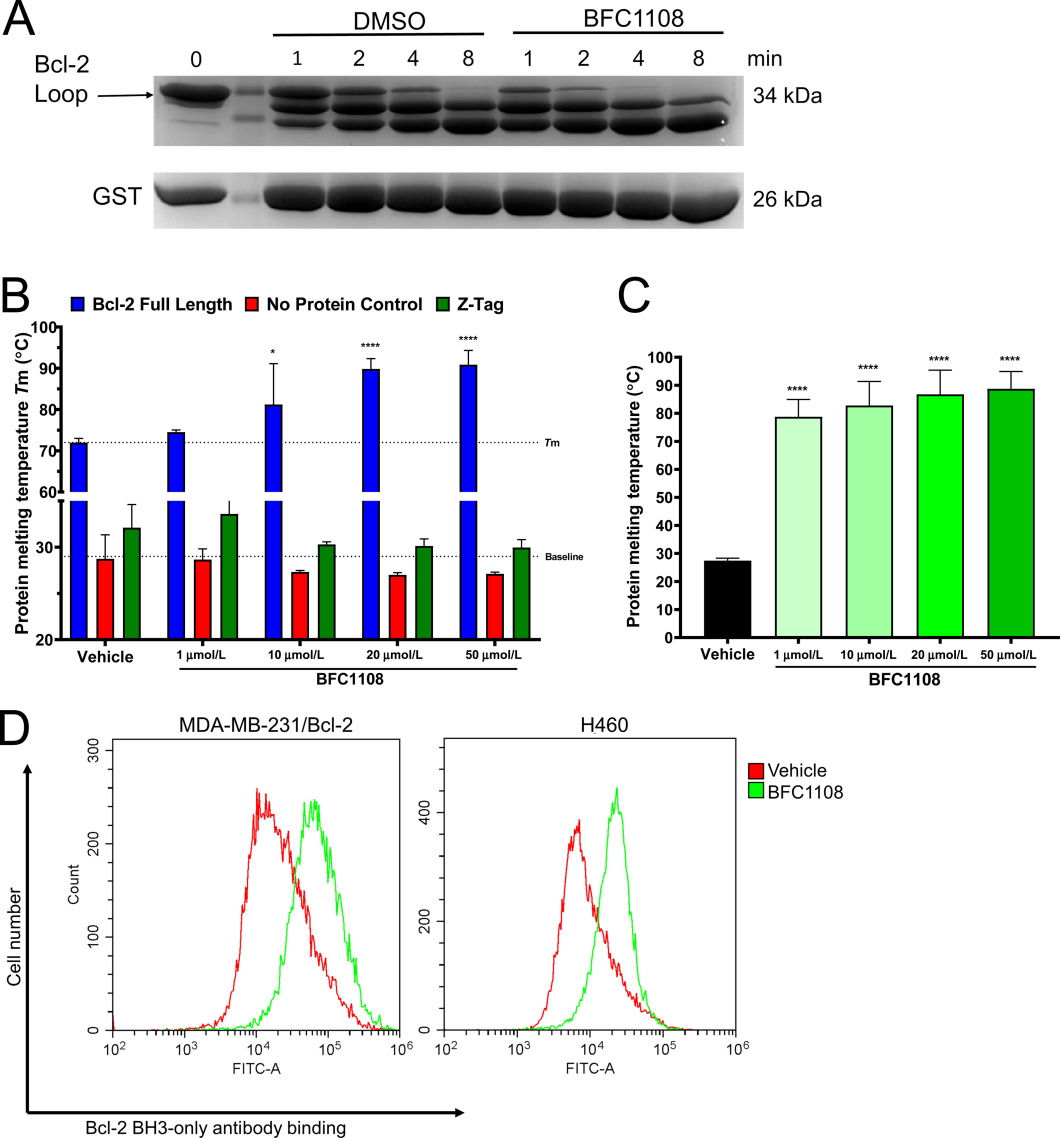
Interaction of BFC1108 with Bcl-2. **A,** Limited proteolysis of Bcl-2 loop domain in the presence of BFC1108. Purified GST-tagged Bcl-2 loop domain and GST only control were incubated with 50 µmol/L BFC1108. The proteolysis pattern of loop domain was determined at the indicated times upon coincubation with trypsin. **B,** BFC1108 stabilizes and increases melting temperature (Tm) of Bcl-2 full-length protein. Thermal unfolding of Bcl-2 full-length protein in the presence of BFC1108 was monitored by SYPRO Orange fluorescence. Z-tag and no protein control samples were included to confirm BFC1108 specific interaction with Bcl-2. Two-way ANOVA with Sidak multiple comparisons *post hoc* test, **, *P* < 0.01; ****, *P* < 0.0001. **C,** BFC1108 stabilizes and increases Bcl-2 loop domain melting temperature (Tm). Thermal unfolding of Bcl-2 loop domain in the presence of BFC1108 monitored by SYPRO Orange fluorescence. One-way ANOVA with Dunnett multiple comparisons *post hoc* test, ****, *P* < 0.0001. **D,** MDA-MB-231/Bcl-2, H460 cells were exposed to BFC1108 at 10 µmol/L concentration for 48 hours in a medium containing 10% serum. Change in conformation of Bcl-2 was determined by using Bcl-2 BH3 antibody followed by flow cytometric analysis.

Nuclear receptor Nur77/TR3 upon translocation from the nucleus or NuBCP-9, a 9 amino acid peptide derived from Nur77, interacts with the loop domain of Bcl-2 and induces Bcl-2–dependent cell death (27, 28, 33, 35). To assess the potential interaction of BFC1108 with Bcl-2, we examined its impact on trypsin-mediated proteolysis of Bcl-2. Our results revealed that BFC1108 increased the proteolysis of the Bcl-2 loop domain compared with the vehicle (Fig. 4A), implying an interaction with the loop domain. To further assess the interaction between Bcl-2 and BFC1108, we purified the Bcl-2 protein and conducted a thermal shift assay. The Bcl-2 full-length protein was incubated with increasing concentrations of BFC1108 or vehicle control and its melting temperature was calculated (46). Bcl-2 full-length protein in the presence of vehicle (DMSO) had a melting point of 73°C and this was stabilized to above 82°C in the presence of BFC1108 (Fig. 4B). To rule out an interaction with the fused Z-tag used for Bcl-2 protein purification, a Z-Tag-only control was employed, showing no change in the melting point temperature (Tm) in the presence of BFC1108. These findings suggest an interaction between BFC1108 and Bcl-2. To explore whether this interaction is specifically with the Bcl-2 loop, the loop domain was expressed as a fusion with the Z-tag, purified, and then subjected to a thermal shift assay after cleaving the Z-tag. The Bcl-2 loop domain was incubated with increasing concentrations of BFC1108, which increased the Bcl-2 loop melting point from 29°C to 78°C, at 1 µmol/L, suggesting an interaction. This melting point remained over 78°C with increased BFC1108 concentrations (Fig. 4C).

NuBCP-9 binding leads to a conformational change in Bcl-2, exposing its BH3 domain and leading to the conversion of Bcl-2 from an anti-apoptotic to a pro-apoptotic protein. This change in conformation of Bcl-2 can be detected by a Bcl-2-BH3 domain antibody. Upon treatment with BFC1108, MDA-MB-231/Bcl-2 cells exhibited the exposure of BH3 domain in Bcl-2, as detected by flow cytometry (Fig. 4D). Similarly, H460 lung cancer cells with high endogenous Bcl-2 expression also showed BH3 domain exposure following treatment with BFC1108 (Fig. 4D) and exhibited responsiveness to BFC1108 (Fig. 2E). Taken together, these findings indicate that BFC1108 likely interacts with the Bcl-2 loop domain, inducing a Bcl-2 conformation change and promoting cell death in Bcl-2–expressing cancer cells.

### Comparison of ABT199, a Small Molecule Inhibitor of Bcl-2 with BFC1108-induced Effects

**FIGURE 5:**
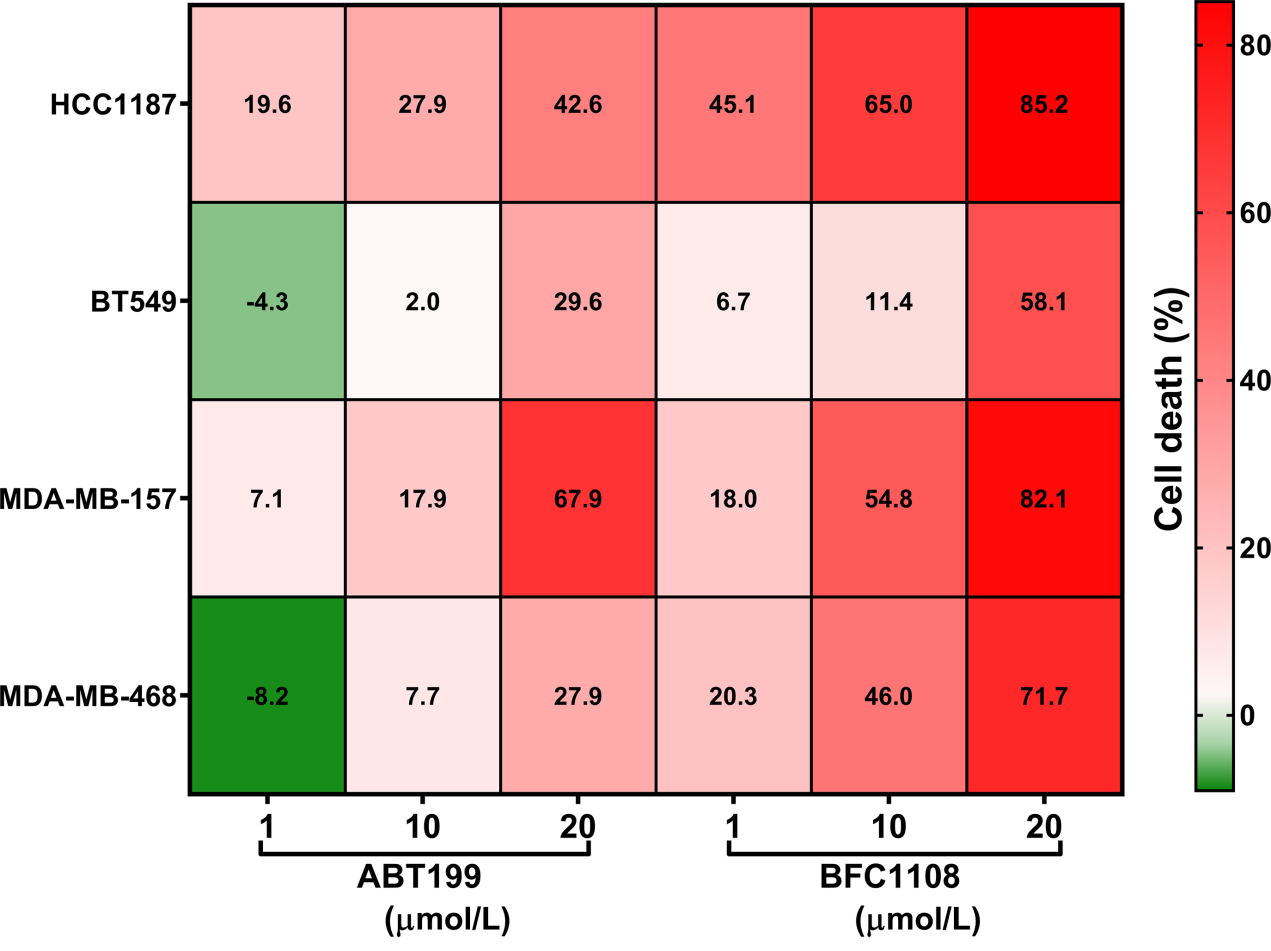
BFC1108 induces cell death across multiple Bcl-2–expressing TNBC cell lines to a greater extent than ABT199. Cell death percentage is calculated based upon viability assay after 72 hours treatment at the indicated BFC1108 concentrations.

We compared the anticancer effects of Bcl-2 functional conversion by BFC1108 to Bcl-2 inhibition through the use of a BH3 mimetic, ABT199 (47). TNBC cell lines that express Bcl-2 (HCC1187, BT549, MDA-MB-157, MDA-MB-468) were treated for 72 hours with BFC1108 or ABT199 and cell death was assessed (Fig. 5). Notably, BFC1108 induced a more substantial level of cell death in HCC1187, MDA-MB-157, and MDA-MB-468 compared with ABT199 (Fig. 5). These findings suggest that Bcl-2 functional conversion may represent a preferable therapeutic strategy in Bcl-2–expressing cancers.

### BFC1108 Suppresses Bcl-2–overexpressing TNBC Xenografts *In Vivo*

**FIGURE 6:**
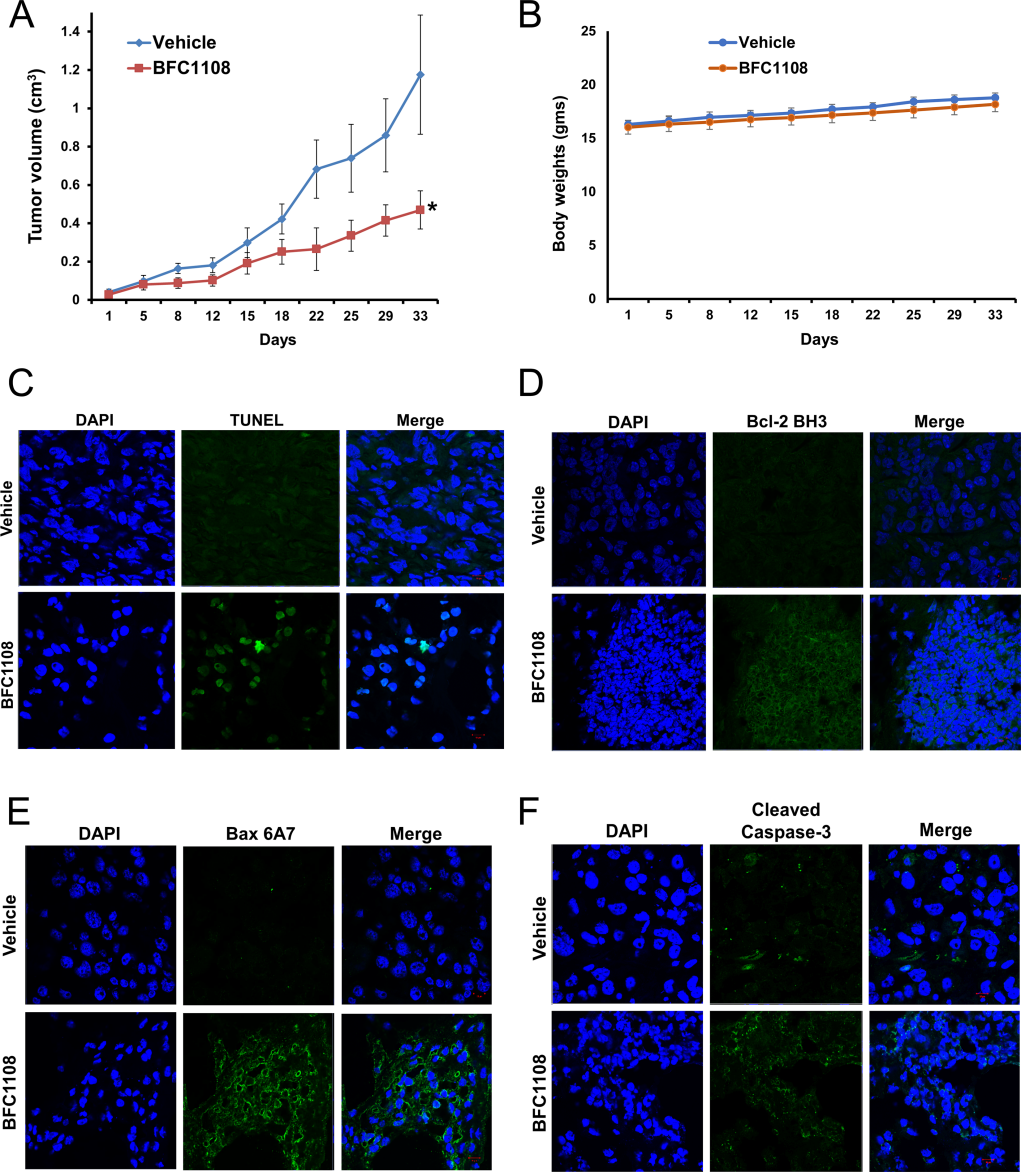
Antitumor effects of BFC1108 in an orthotopic breast cancer model. **A,** BFC1108 inhibits growth of high Bcl-2–expressing MDA-MB-231 TNBC cells *in vivo*. A total of 10^6^ MDA-MB-231/Bcl-2 cells were implanted in the mammary fat pad of NOD.SCID mice (*n* = 8 per group). Once palpable tumors were detected, mice were randomized and treated with vehicle or BFC1108 at 100 mg/kg twice a week by intraperitoneal route. Tumor measurements were made with digital calipers twice a week. *, *P* < 0.05 from day 18 onward. **B,** The body weights of mice treated with vehicle or BFC1108 in A were measured twice a week. **C,** Induction of apoptosis of tumor cells by BFC1108 *in vivo*. Immunofluorescence was performed on frozen tumor sections to determine apoptosis by TUNEL staining. **D,** BFC1108 induces conformation change of Bcl-2 in tumor tissues. Bcl-2 conformational change was detected by staining with Bcl-2 BH3 antibody. **E,** Bax activation by BFC1108. Activated Bax was detected using Bax 6A7 antibody in tumor tissues from animals treated with vehicle or BFC1108. **F,** Activation of caspase-3 by BFC1108. Activated caspase-3 was detected using cleaved caspase-3 antibody in tumor tissues from animals treated with vehicle or BFC1108.

To evaluate the *in vivo* effectiveness of BFC1108, mice bearing orthotopic breast cancer xenografts originating from high Bcl-2–expressing MDA-MB-231 cells (MDA-MB-231/Bcl-2) underwent a 4-week treatment administered via the intraperitoneal route. The administration of BFC1108 led to a significant reduction in the growth of MDA-MB-231/Bcl-2 xenograft tumors (Fig. 6A), without causing a substantial change in body weight of mice (Fig. 6B). We examined tumor sections for apoptosis by staining for TUNEL and active caspase-3. The detection of TUNEL positive cells provided confirmation of BFC1108-induced apoptosis *in vivo* (Fig. 6C). In addition, we investigated these tumor sections to detect changes in Bcl-2 conformation (Fig. 6D). In the tumor sections from mice treated with BFC1108, we observed an exposure of the Bcl-2 BH3 domain (Fig. 6D). To establish a connection between the altered Bcl-2 conformation, the activation of Bax, and the induction of apoptosis, we probed the tumor sections with antibodies that recognize activated Bax and cleaved (active) caspase-3 (Fig. 6E and F). Bax activation was detected in tumor sections from mice treated with BFC1108, along with the presence of activated caspase-3. Thus, there is a correlation between exposure of BH3 domain in Bcl-2, activation of Bax, and the induction of apoptosis *in vivo*.

### BFC1108 Suppresses Breast Cancer Lung Metastasis *In Vivo*

**FIGURE 7:**
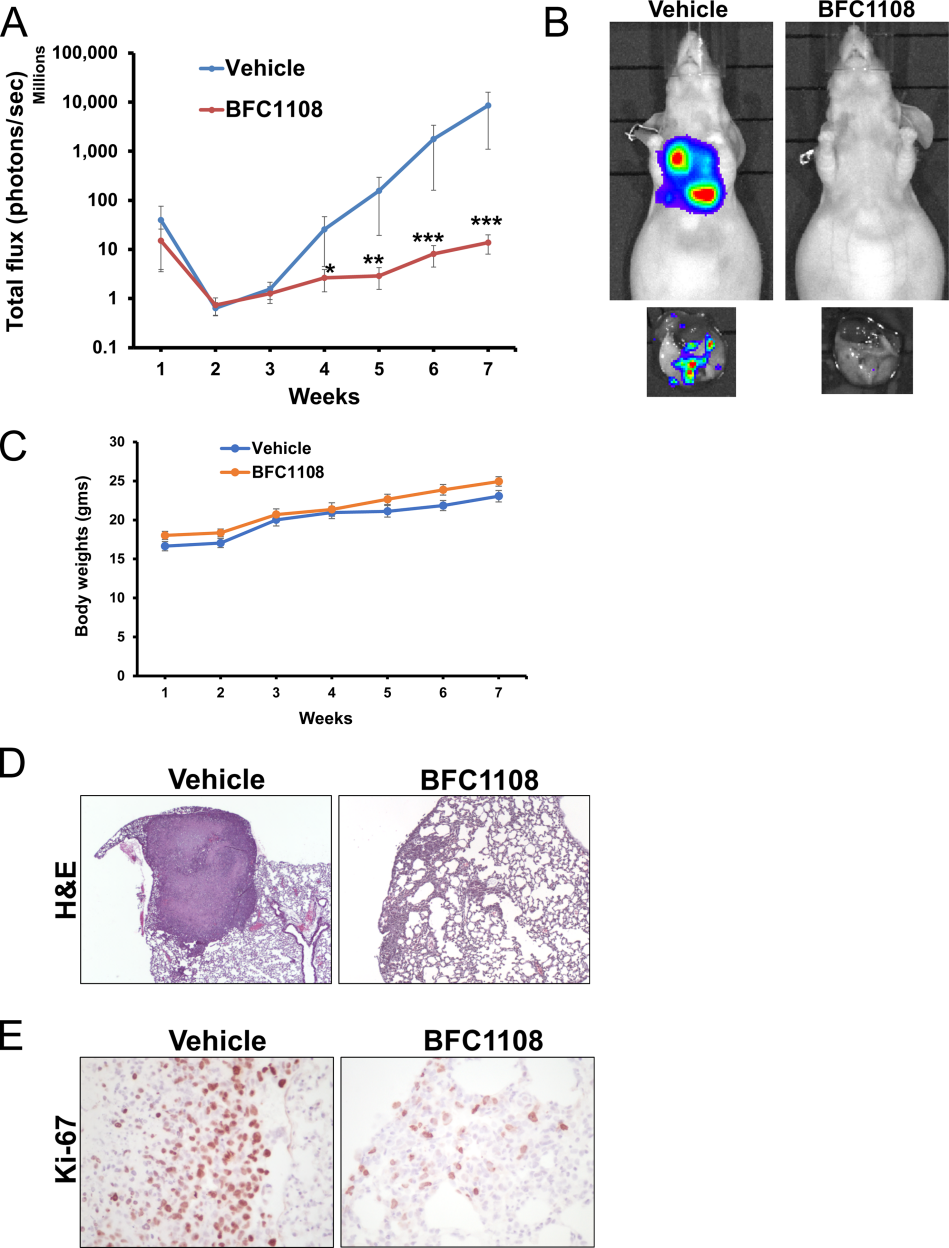
Inhibition of breast cancer lung metastasis by BFC1108. **A,** BFC1108 suppresses the growth of lung metastasis *in vivo*. LMD-231 cells (200,000) stably expressing luciferase were injected into the tail vein of 6-week-old nude mice. Lung metastasis was detected in 2 weeks. Mice were randomized and were treated four times a week with vehicle or 100 mg/kg BFC1108 by intraperitoneal route (*n* = 8 for vehicle treatment and *n* = 9 for BFC1108 treatment). Bioluminescent imaging was performed once a week and quantified. *, *P* < 0.05; **, *P* < 0.01; ***, *P* < 0.001. Bioluminescent imaging data for individual mouse treated with vehicle or BFC1108 are shown in Supplementary Fig. S2. **B,** Representative mouse image from vehicle and BFC treatment is shown. **C,** Treatment with BFC1108 does not alter body weight of mice. Body weights were measured each time the mice were imaged. **D,** Suppression of lung metastasis by BFC1108. H&E staining showed increased number of tumor cells in lung tissue of mice with vehicle treatment, compared with BFC1108 treatment, at the end of the study. **E,** BFC1108 reduces proliferation of lung metastatic TNBC cells. Detection of Ki-67 indicating proliferating tumor cells in lung tissue from vehicle and BFC1108-treated mice.

We next tested the efficacy of BFC1108 in suppressing breast cancer lung metastasis. LMD231 cells are lung metastatic cells derived from TNBC MDA-MB-231 cells (48, 49). LMD231 cells were engineered to stably express luciferase (LMD231-Luc) to aid in the detection of metastasis. Following injection through the tail vein, LMD231-Luc cells formed lung metastases within 2 weeks. Mice were treated with 100 mg/kg of BFC1108, four times a week via intraperitoneal injections. After 7 weeks, BFC1108 significantly suppressed the growth of lung metastasis (Fig. 7A and B; Supplementary Fig. S2). There was no significant change in body weight of mice during the course of the treatment (Fig. 7C). The reduction in lung metastasis was independently confirmed through histologic analysis (Fig. 7D). IHC of the lung tissues from vehicle-treated animals revealed Ki-67 positivity, indicating presence of actively proliferating tumor cells. In contrast, Ki-67 staining was dramatically reduced in lung tissues from BFC1108-treated mice (Fig. 7E).

## Discussion

In this study, we identified BFC1108, a small molecule Bcl-2 functional converter, that suppresses primary and metastatic breast cancers in mouse xenograft models. Bcl-2 and its family members are prime molecular targets for developing new cancer therapeutics (3, 6, 14, 17, 18). There have been several efforts in development of Bcl-2 inhibitors for solid cancers, but not with very encouraging results in clinical trials (18, 22, 24). Our study provides a proof of concept for the therapeutic targeting of Bcl-2 through the use of small molecules that induce the Bcl-2 killer conformation.

BFC1108 induces apoptosis in cancer cells expressing Bcl-2. There is also a correlation between the extent of Bcl-2 expression and responsiveness of cancer cells to BFC1108. This may be due to the interaction of BFC1108 with the loop domain of Bcl-2 resulting in exposure of its BH3 domain and conversion into a pro-apoptotic protein. The interaction between BFC1108 and Bcl-2 was demonstrated through thermal shift and limited proteolysis assays. Similar to NuBCP-9, BFC1108-induced apoptosis is also dependent on Bax and/or Bak. More importantly, BFC1108 suppressed xenograft growth of human breast cancer cells with high Bcl-2 expression in mice, without any systemic toxicity. The apoptotic effect of BFC1108 is not inhibited, but rather potentiated, by Bcl-2 expression in multiple cancer cell lines. BFC1108 also suppressed the growth of breast cancer lung metastasis, underscoring its potential for treating metastatic disease. Thus, BFC1108 is a promising clinical candidate for treating both primary and metastatic cancers expressing Bcl-2. Notably, BFC1108 exhibits significantly better efficacy in TNBC cells expressing Bcl-2 compared with a Bcl-2 inhibitor, ABT199.

The ability to target and kill Bcl-2–expressing cells is exceptionally promising, as Bcl-2 is overexpressed in several cancers including TNBC. Selective targeting of Bcl-2 through an unexplored region and elimination of cancer cells expressing Bcl-2 open up new therapeutic possibilities. “Bcl-2 functional converters” are envisioned to function as targeted cancer therapeutics, exploiting the cancer-specific overexpression of Bcl-2.

## Supplementary Material

Supplemental Figure 1 Bcl-2 dependent apoptotic effects of BFC1108. Independent experimental replicates of data in Figure 2C are shown.

Supplemental Figure 2 Bioluminescent imaging of data in Figure 7 for individual mouse treated with vehicle or BFC1108 is shown.
