# Supplementary material for: Identification and Characterization of a Small Molecule Bcl-2 Functional Converter
Source: Cancer Res Commun. 2024 Mar 4;4(3):634–44. doi: 10.1158/2767-9764.CRC-22-0526 (PMC10911799; doi:10.1158/2767-9764.CRC-22-0526)
Supplement: Supplemental Figure 1 — Bcl-2 dependent apoptotic effects of BFC1108. Independent experimental replicates of data in Figure 2C are shown. [file crc-22-0526-s01.pdf]

Supplemental Figure 1

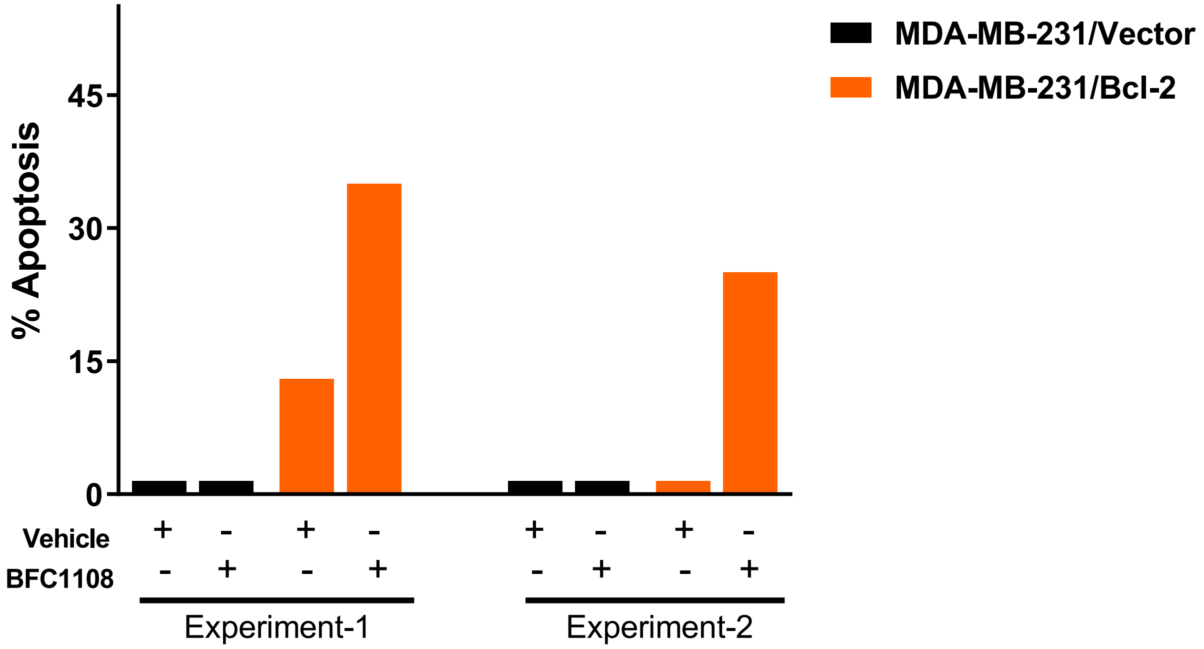

Supplemental Figure 1: Bcl-2 dependent anti-tumorigenic effects of BFC1108.

Independent experimental replicates of data in Figure 2C are shown.
