# Supplementary material for: Identification and Characterization of a Small Molecule Bcl-2 Functional Converter
Source: Cancer Res Commun. 2024 Mar 4;4(3):634–44. doi: 10.1158/2767-9764.CRC-22-0526 (PMC10911799; doi:10.1158/2767-9764.CRC-22-0526)
Supplement: Supplemental Figure 2 — Bioluminescent imaging of data in Figure 7 for individual mouse treated with vehicle or BFC1108 is shown. [file crc-22-0526-s02.pdf]

Supplemental Figure 2

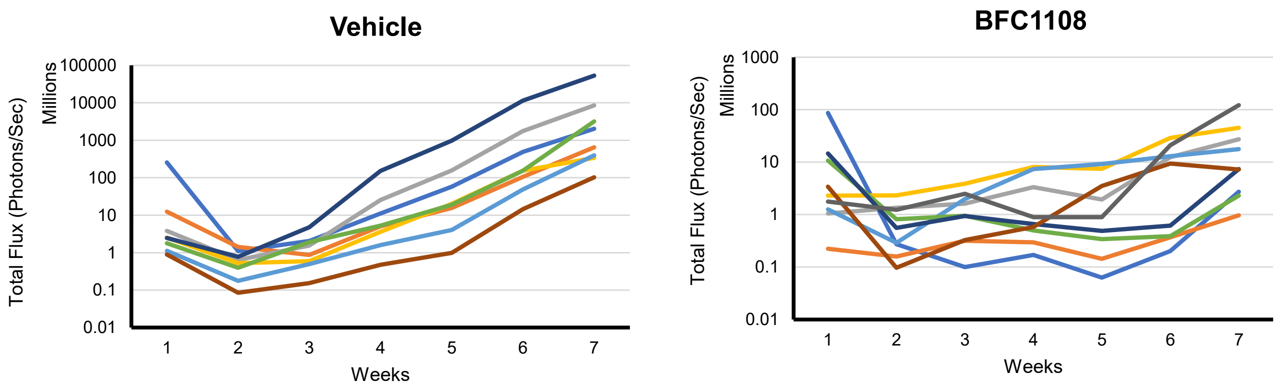

Supplemental Figure 2: Bioluminescent imaging data in Figure 7 for individual mouse treated with vehicle or BFC1108 is shown.
